# Supplementary material for: Synbiotics Alleviate Hepatic Damage, Intestinal Injury and Muscular Beclin-1 Elevation in Rats after Chronic Ethanol Administration
Source: Int J Mol Sci. 2021 Nov 21;22(22):12547. doi: 10.3390/ijms222212547 (PMC8622351; doi:10.3390/ijms222212547)
Supplement: Supplementary file 1 [file ijms-22-12547-s001.zip › ijms-1433295-supplementary.pdf]

Supplementary Table S1. Effects of synbiotics supplementation on plasma amino acid composition in rats with chronic alcohol feeding[1].

nM	C		E		SC		PSE		ASE	
Valine	245.19 ± 41.58		423.52 ± 196.93		228.22 ± 49.80		347.22 ± 251.22		400.15 ± 216.08	
Isoleucine	315.18 ± 20.20		360.62 ± 32.57		335.44 ± 119.90		290.68 ± 31.11		329.83 ± 53.60	
Leucine	320.13 ± 18.69	ab	478.34 ± 127.03	a	294.63 ± 49.96	b	370.45 ± 108.23	ab	362.15 ± 56.41	ab
ΣBCAAs	880.50 ± 55.20		1262.48 ± 312.64		858.30 ± 211.11		1008.36 ± 329.78		1092.13 ± 237.87	
Phenylalanine	313.07 ± 89.79	b	514.39 ± 13.56	a	330.12 ± 68.80	b	249.97 ± 22.25	b	372.44 ± 123.46	b
Tryptophan	33.35 ± 5.56		38.27 ± 0.38		37.19 ± 1.70		33.90 ± 4.71		36.19 ± 1.89	
Tyrosine	158.00 ± 17.86	ab	191.25 ± 5.97	a	160.96 ± 24.58	ab	131.91 ± 31.54	b	166.03 ± 11.50	ab
ΣAAAs	504.43 ± 102.50	b	743.92 ± 11.91	a	528.28 ± 82.42	b	415.79 ± 43.81	b	574.66 ± 134.20	b
Methionine	20.97 ± 5.31	b	35.77 ± 5.30	a	17.75 ± 13.57	b	4.54 ± 1.50	c	21.58 ± 4.03	b
Histidine	1.13 ± 1.96	b	719.03 ± 549.24	a	132.30 ± 229.15	b	0.00 ± 0.00	b	15.47 ± 26.79	b
Alanine	6.40 ± 11.09	b	52.80 ± 34.01	a	0.00 ± 0.00	b	16.80 ± 8.05	b	15.00 ± 13.85	b
Arginine	2.95 ± 0.74	a	1.10 ± 0.36	b	1.02 ± 0.09	b	0.68 ± 0.72	b	1.21 ± 0.46	b
Proline	42.29 ± 12.12	b	59.41 ± 6.96	a	52.36 ± 2.12	ab	50.37 ± 3.19	ab	53.63 ± 3.99	ab
Lysine	4.93 ± 2.53		7.00 ± 1.74		6.44 ± 0.38		4.76 ± 1.17		5.18 ± 1.09	
Glutamine	7.76 ± 0.20	a	6.81 ± 0.53	ab	6.05 ± 0.53	b	4.48 ± 0.68	c	4.18 ± 1.25	c
Glutamic acid	2.78 ± 0.47		2.82 ± 0.39		2.80 ± 0.08		3.14 ± 0.54		2.98 ± 0.28	
Aspartic acid	0.20 ± 0.14		0.24 ± 0.14		0.22 ± 0.24		0.29 ± 0.22		0.02 ± 0.04	
Asparagine	0.00 ± 0.00		0.00 ± 0.00		0.00 ± 0.00		0.00 ± 0.00		0.00 ± 0.00	
Threonine	0.00 ± 0.00		0.11 ± 0.19		0.03 ± 0.05		0.00 ± 0.00		0.49 ± 0.85	
Serine	0.00 ± 0.00		0.00 ± 0.00		0.00 ± 0.00		0.00 ± 0.00		0.00 ± 0.00	

[1] Data are expressed as the mean ± standard deviation ($n = 6$). Different letters (a, b, and c) indicate a significant difference at $p < 0.05$ by a one-way ANOVA with Duncan's post-hoc test.

Supplementary Table S2. Effects of synbiotics supplementation on hepatic amino acid composition in rats with chronic alcohol feeding[1].

mM/g protein	C		E		SC		PSE		ASE	
Valine	47.38 ± 18.61		41.45 ± 10.03		38.32 ± 9.60		32.62 ± 2.71		44.07 ± 10.12	
Isoleucine	55.71 ± 16.30	a	51.88 ± 7.97	ab	43.10 ± 10.17	ab	33.53 ± 6.20	ab	25.85 ± 23.40	b
Leucine	63.41 ± 30.60	a	42.30 ± 16.15	ab	35.38 ± 11.20	ab	16.12 ± 3.17	b	19.03 ± 14.76	b
ΣBCAAs	166.50 ± 48.43	a	135.63 ± 24.98	ab	116.80 ± 29.96	ab	82.27 ± 11.76	b	88.95 ± 34.40	b
Phenylalanine	54.47 ± 18.37	a	45.48 ± 7.63	ab	34.59 ± 8.33	b	30.16 ± 2.02	b	38.06 ± 3.81	ab
Tryptophan	1.44 ± 0.45	a	1.18 ± 0.14	ab	1.13 ± 0.27	ab	0.81 ± 0.07	b	1.00 ± 0.08	ab
Tyrosine	40.00 ± 11.25	a	36.44 ± 6.34	ab	28.44 ± 6.95	ab	25.05 ± 2.74	b	30.82 ± 3.09	ab
ΣAAAs	95.92 ± 29.40	a	83.11 ± 14.10	ab	64.16 ± 15.53	ab	56.03 ± 4.76	b	69.88 ± 6.84	ab
Methionine	14.16 ± 11.37		9.91 ± 4.98		16.96 ± 2.35		8.80 ± 1.43		13.04 ± 4.26	
Histidine	2.70 ± 0.96	a	2.20 ± 0.22	ab	0.82 ± 1.08	b	1.57 ± 0.19	ab	1.33 ± 1.07	ab
Alanine	0.24 ± 0.04	ab	0.34 ± 0.07	a	0.21 ± 0.07	b	0.18 ± 0.03	b	0.29 ± 0.07	ab
Arginine	0.07 ± 0.02		0.04 ± 0.01		0.06 ± 0.03		0.04 ± 0.01		0.04 ± 0.01	
Proline	11.97 ± 2.88	b	21.65 ± 5.87	a	20.10 ± 4.90	ab	14.72 ± 1.00	ab	20.86 ± 2.62	ab
Lysine	4.18 ± 3.87		3.53 ± 1.92		2.92 ± 1.06		1.80 ± 0.62		1.70 ± 0.67	
Glutamine	5.27 ± 3.08	a	2.43 ± 0.36	ab	2.73 ± 1.31	ab	2.18 ± 0.28	b	2.22 ± 0.10	b
Glutamic acid	3.56 ± 0.15	a	2.06 ± 0.49	b	2.77 ± 0.19	ab	2.08 ± 0.41	b	2.54 ± 0.81	b
Aspartic acid	5.54 ± 2.84		5.88 ± 2.73		4.74 ± 1.11		4.68 ± 1.53		5.77 ± 2.12	
Asparagine	0.00 ± 0.00		0.00 ± 0.00		0.00 ± 0.00		0.00 ± 0.00		0.00 ± 0.00	
Threonine	6.41 ± 7.19		2.59 ± 2.46		3.28 ± 2.62		0.92 ± 0.70		2.97 ± 0.22	
Serine	0.0000 ± 0.0000		0.0000 ± 0.0000		0.0009 ± 0.0015		0.0000 ± 0.0000		0.0008 ± 0.0013	

[1] Data are expressed as the mean ± standard deviation (n = 6). Different letters (a and b) indicate a significant difference at $p < 0.05$ by a one-way ANOVA with Duncan's post-hoc test.

Supplementary Table S3. Effects of synbiotics on supplementation on muscle amino acid composition in rats with chronic alcohol feeding[1].

mM/g protein	C	E	SC	PSE	ASE
Valine	18.18 ± 17.30	30.52 ± 5.85	48.06 ± 28.55	24.57 ± 7.16	20.38 ± 8.53
Isoleucine	19.32 ± 6.13	50.74 ± 30.80	62.66 ± 37.97	24.87 ± 8.42	47.51 ± 19.61
Leucine	34.16 ± 13.70	45.13 ± 10.10	48.67 ± 21.56	34.31 ± 10.15	66.76 ± 27.46
ΣBCAAs	71.65 ± 30.63	126.39 ± 39.23	159.39 ± 80.58	83.75 ± 20.33	134.65 ± 36.85
Phenylalanine	33.64 ± 23.88 [b]	65.79 ± 21.29 [ab]	88.38 ± 27.22 [a]	51.57 ± 23.39 [ab]	46.65 ± 17.13 [ab]
Tryptophan	1.29 ± 0.69	1.48 ± 0.85	2.02 ± 0.64	1.14 ± 0.42	2.35 ± 1.87
Tyrosine	19.27 ± 8.88	33.87 ± 11.16	64.67 ± 57.31	18.85 ± 7.18	37.40 ± 11.36
ΣAAAs	54.20 ± 30.47 [b]	101.14 ± 27.49 [ab]	155.08 ± 84.68 [a]	71.56 ± 30.96 [ab]	86.39 ± 22.36 [ab]
Methionine	2.90 ± 1.69 [b]	5.29 ± 0.80 [ab]	9.58 ± 5.16 [a]	4.49 ± 1.71 [ab]	5.73 ± 1.75 [ab]
Histidine	0.29 ± 0.28	1.38 ± 1.14	1.30 ± 1.02	0.72 ± 0.22	1.25 ± 0.69
Alanine	0.18 ± 0.17	0.19 ± 0.15	0.30 ± 0.14	0.13 ± 0.03	0.43 ± 0.32
Arginine	0.20 ± 0.06	0.27 ± 0.22	0.45 ± 0.14	0.22 ± 0.11	0.38 ± 0.21
Proline	3.29 ± 2.32	5.28 ± 1.81	5.64 ± 2.55	3.28 ± 1.79	4.03 ± 2.03
Lysine	0.39 ± 0.11 [b]	0.51 ± 0.35 [ab]	1.10 ± 0.52 [a]	0.25 ± 0.08 [b]	0.77 ± 0.33 [ab]
Glutamine	0.37 ± 0.22	0.29 ± 0.33	0.38 ± 0.35	0.22 ± 0.04	0.43 ± 0.24
Glutamic acid	1.38 ± 0.99 [b]	2.51 ± 0.97 [b]	5.58 ± 2.81 [a]	1.76 ± 0.51 [b]	1.99 ± 1.16 [b]
Aspartic acid	0.11 ± 0.05 [ab]	0.01 ± 0.02 [b]	0.27 ± 0.26 [a]	0.09 ± 0.03 [ab]	0.06 ± 0.05 [ab]
Asparagine	0.00 ± 0.00	0.00 ± 0.00	0.00 ± 0.00	0.00 ± 0.00	0.00 ± 0.00
Threonine	0.0000 ± 0.0000	0.0000 ± 0.0000	0.0087 ± 0.0150	0.0000 ± 0.0000	0.1259 ± 0.2181
Serine	0.00 ± 0.00	0.00 ± 0.00	0.00 ± 0.00	0.00 ± 0.00	0.00 ± 0.00

[1] Data are expressed as the mean ± standard deviation (n = 6). Different letters (a and b) indicate a significant difference at $p < 0.05$ by a one-way ANOVA with Duncan's post-hoc test.
